# *EGFR*野生型晚期非小细胞肺癌的治疗进展

**DOI:** 10.3779/j.issn.1009-3419.2014.07.14

**Published:** 2014-07-20

**Authors:** 峰 马, 晓宇 史, 玮 孟, 敬 张, 丽霞 赵, 晓华 何, 峻峰 赵

**Affiliations:** 075000 张家口，河北北方学院附属第一医院肿瘤内科 Department of Medical Oncology, the First Affiliated Hospital of Hebei North University, Zhangjiakou 075000, China

**Keywords:** 肺肿瘤, 表皮生长因子受体, 野生型, 化疗, 靶向治疗, 维持治疗, Lung neoplasms, Epidermal growth factor receptor, Wild type, Chemotherapy, Targeted therapy, Maintenance treatment

## Abstract

肺癌是癌症死亡的主要原因。肺癌的治疗仍是医疗界最具挑战性的任务之一。表皮生长因子受体酪氨酸激酶抑制剂（epidermal growth factor receptor-tyrosine kinase inhibitor, EGFR-TKI）的发现和发展，对非小细胞肺癌（non-small cell lung cancer, NSCLC）的治疗产生了重大影响。但EGFR-TKI对*EGFR*野生型NSCLC的疗效较差，而有限的EGFR突变率也促使研究者们不断探索*EGFR*野生型NSCLC的最佳的治疗选择。本文将对*EGFR*野生型NSCLC的治疗现状进行综述。

## Abstract (en)

Lung cancer is the leading cause of death from cancer in the world. The treatment remains one of the most challenging tasks in the medical world. The discovery and development of epidermal growth factor receptor-tyrosine kinase inhibitor (EGFR-TKI) have had a major impact in the treatment of non small cell lung cancer (NSCLC). But the efficacy of EGFR-TKI in *EGFR* wild-type patients is limited, and the limited *EGFR* mutation incidence also prompts researchers to study what is the best treatment choice for patients with NSCLC who are negative for *EGFR* mutations. This review will discuss the research status in treatment choice for *EGFR* wild-type NSCLC.

肺癌是全世界最常见的恶性肿瘤之一，在过去的几十年里，肺癌正逐渐成为全世界发病率和死亡率最高的肿瘤。肺癌主要分为小细胞肺癌和非小细胞肺癌（non -small cell lung cancer, NSCLC），其中NSCLC约占85%，NSCLC可进一步分为非鳞状细胞癌（腺癌、大细胞肺癌和其他细胞类型）和鳞癌。但每一位肺癌患者在临床特征、预后、治疗反应和耐受性等方面都是独特的，因此寻找能预测患者治疗获益的标志物成为研究的重点。2004年4月-5月，在*The New England Journal of Medicine*和*Science*上发表了两篇关于“表皮生长因子受体（epidermal growth factor receptor, *EGFR*）基因突变预测酪氨酸激酶抑制剂治疗肺癌敏感性”的文章^[1, 2]^。自此，对于不同组织类型的肺癌可进行进一步的分子分型。从此，肺癌的治疗走上了以基因为靶点的个体化治疗之路。目前研究表明，*EGFR*突变阳性（主要发生在第19和21外显子）的NSCLC，应用EGFR酪氨酸激酶抑制剂（EGFR-tyrosine kinase inhibitor, EGFR-TKI）单药治疗安全有效，其代表药物厄洛替尼已经成为*EGFR*突变阳性NSCLC的标准一线治疗方案，而对EGFR野生型的NSCLC，EGFR-TKI的治疗效果欠佳^[3]^。那么对于EGFR野生型的NSCLC该如何治疗呢？

## 1 *EGFR*野生型NSCLC的临床病理学特点及检测方法

*EGFR*突变情况对于治疗方案的选择具有重要意义，因此掌握*EGFR*野生型NSCLC的临床病理特点和*EGFR*突变检测方法显得尤为重要。

### 1.1 临床病理特点

临床中相当一部分患者由于不能手术，或没有足够的病理取材，或因费用问题不能进行突变状态的检测。因此很多学者希望找到与突变状态相关的临床病理学特点。目前研究表明：*EGFR*野生型在鳞癌中少见，在腺癌中所占比例较突变者高，且以腺泡癌亚型为主^[4]^。野生型患者还具有男性、吸烟、临床分期较晚、胸部计算机断层扫描（computed tomography, CT）中肿瘤最大径更大，且多呈实体阴影，正电子发射型计算机断层扫描-CT（positron emission tomography-CT, PET-CT）中具有更高的最高标准吸收值等特点^[5]^。这些特点可以帮助我们对不能确定突变状态的患者做出更准确的判断，从而实施更佳的诊疗选择。

### 1.2 检测方法

组织标本作为*EGFR*突变检测的材料准确可靠，但因组织量不足或部位不理想而有一定局限性。细胞学样本，如恶性胸腔积液和细针穿刺，也适用于*EGFR*突变测试，但对检测方法的灵敏度要求较高。外周血*EGFR*突变检测取材简便，创伤性小，与组织学检测有较高的符合率，并能动态监测*EGFR*突变状态，有望对组织学检测进行补充。

检测方法中直接测序法是*EGFR*突变检测的“金标准”，也是使用最多的方法。但是测序法操作复杂，费用昂贵，灵敏度不高，因此，在临床应用中还存在一定的限制^[6]^。其他常用的检测方法有聚合酶链式反应-单链构象多态性分析、探针扩增阻滞突变系统（amplification refractory mutation system, ARMS）、突变体富集PCR（mutant-enriched polymerase chain reaction）、高效液相色谱法（denaturing high-performance liquid chromatograph, DHPLC）、高分辨率溶解曲线分析技术（high resolution melting analysis, HRM）等，这些方法灵敏性高，快速，操作简单。其中ARMS、DHPLC、mutant-enriched PCR有报道可以用于外周血*EGFR*突变的检测。

## 2 *EGFR*野生型NSCLC的治疗选择

### 2.1 一线治疗选择

#### 2.1.1 化疗

铂类为基础的双药联合化疗仍是*EGFR*野生型患者的首选。目前研究表明对于非鳞状细胞癌更倾向于使用顺铂或卡铂/培美曲塞作为一线治疗方案，而吉西他滨/顺铂则更适用于鳞癌。因此人们也试图寻找*EGFR*突变状态与化疗之间的联系。

*EGFR*突变状态与化疗有着怎样的关系呢？既往有研究认为*EGFR*突变阳性NSCLC一线化疗效果优于野生型^[7, 8]^。但这些研究均未进一步对不同一线化疗方案亚组进行区分。那么*EGFR*突变状态是否对不同化疗方案的选择具有预测作用呢？

朱军等^[9]^的研究显示，当患者接受吉西他滨或长春瑞滨/铂类的一线方案化疗时，*EGFR*野生型患者的无进展生存期（progression free survival, PFS）和总生存期（overall survival, OS）均较*EFGR*突变阳性患者有延长，但OS的延长更有实际意义（9.2个月 *vs* 7.8个月，*P*=0.028）。另一项研究^[10]^显示，不同的突变状态患者，在接受以培美曲塞为基础的一线方案化疗时，生存无统计学差异。Park等^[11]^研究认为，*EGFR*突变阳性患者接受以多西他赛为基础的含铂双药方案时，相比吉西他滨，能够获得更长的PFS（5.3个月 *vs* 3.7个月，*P*=0.012），而野生型患者无此差异。董晓鹏等^[12]^对229例接受多以西他赛或长春瑞滨或吉西他滨/卡铂为一线方案化疗的NSCLC进行分析得出相似结论，研究认为*EGFR*突变阳性组PFS较野生型组明显延长（8.3个月 *vs* 9.1个月，*P*=0.008），阳性组中多西他赛或长春瑞滨组较吉西他滨组PFS更有优势（9.4个月 *vs* 8.3个月，*P*=0.033；9.6个月 *vs* 8.3个月，*P*=0.028），*EGFR*野生型组中无此差异。

以上分析表明，*EGFR*突变状态与化疗疗效相关，且对化疗方案的选择可能具有一定预测作用，但目前对于*EGFR*野生型化疗选择的循证医学证据仍较低，期待着大规模前瞻性临床试验的进一步证实。

#### 2.1.2 靶向治疗

EGFR-TKI与化疗孰优孰劣一直是人们希望回答的问题，近日Lee等^[3]^发表在《美国医学会杂志》上的一项荟萃分析表明，在*EGFR*野生型患者中，与EGFR-TKI相比，化疗组PFS和客观缓解率（objective response rate, ORR）更具优势，但OS无明显差异。这项研究表明，对于*EGFR*野生型患者，化疗可能会是一个比EGFR-TKI更好的治疗选择，尽管这项建议不是结论性的，因为总体比较并非基于随机化的试验。但这是否意味着化疗是野生型患者的唯一选择呢？由于*EGFR*突变往往与其他基因变异相互排斥，因此有必要寻找*EGFR*野生型患者中存在的分子亚型，进而为其提供更有针对性的个体化靶向治疗^[13]^。过去的十年里，许多新靶点发现和新的治疗策略的发展，为*EGFR*野生型患者的治疗带来更多的选择。

间变性淋巴瘤激酶（anaplastic lymphoma kinase, *ALK*）基因重排的发现，进一步强化了靶向治疗在NSCLC治疗中的作用。在肺癌患者中*ALK*基因重排的比例约为2%-7%，但在具有更加年轻、男性、不吸烟或少量吸烟、腺癌等临床特点的NSCLC患者中，*ALK*基因重排可以占到30%^[14, 15]^。*ALK*基因重排与*EGFR*及*KRAS*突变相互排斥，与*EGFR*野生型独立相关，在*EGFR*和*KRAS*均为野生型的肺腺癌患者中*ALK*基因重排阳性率可高达30%-42.8%^[16, 17]^。克唑替尼是一种ALK及MET小分子抑制剂。基于两项（PROFILE 1001、1005）共纳入255例局部晚期或转移的ALK阳性NSCLC患者的Ⅰ期/Ⅱ期临床试验的安全性和有效性数据，2011年8月美国食品及药物管理局（Food and Drug Administration, FDA）批准该药用于ALK阳性的局部晚期或转移的NSCLC患者。更新的数据^[18]^表明，克唑替尼在携带ALK重排以及既往治疗后进展的晚期NSCLC患者中治疗反应率高达60.8%，中位PFS为9.7个月，6个月及12个月生存率分别为87.9%和74.8%。目前比较克唑替尼与标准的一线化疗(培美曲塞/顺铂或卡铂)的两项Ⅲ期随机开放的临床试验（PROFILE 1014和NCT01639001）正在研究中。

EGFR是原癌基因*c*-*erbB1*的表达产物，是表皮生长因子受体家族成员之一，与肿瘤细胞的增殖、血管生成、肿瘤侵袭、转移及细胞凋亡的抑制有关。西妥西单抗是一种嵌合的抑制EGFR通路的单克隆抗体。一项大型Ⅲ期随机试验（FLEX）对顺铂/长春瑞滨加或不加西妥昔单抗用于晚期NSCLC进行了评估：西妥昔单抗组缓解率提高（36% *vs* 29%, *P*=0.012），PFS无差别，但接受西妥昔单抗患者的OS更长（11.3个月 *vs* 10.1个月，*P*=0.04）^[19]^。O'Byrne等^[20]^对FLEX实验中的样本进行*EGFR*突变检测发现，该试验中85%为*EGFR*野生型，且突变状态与疗效无相关性。因此美国国立综合癌症网络指南（National Comprehensive Cancer Network, NCCN）推荐西妥昔单抗/顺铂/长春瑞滨可作为无*EGFR*突变晚期NSCLC的一种治疗选择。

血管内皮生长因子（vascular endothelial growth factor, VEGF）是诱导肿瘤血管形成作用较强和特异的调节因子，它参与肺癌血管的再生、肿瘤的生长和转移。贝伐单抗作为一种针对VEGF的重组单克隆抗体，2006年被FDA批准用于不可切除、局部晚期、复发或转移的非鳞状细胞癌。美国东部肿瘤协作组推荐贝伐单抗联合紫杉醇/卡铂用于晚期非鳞状细胞癌，依据为一项大型Ⅲ期临床试验ECOG 4599的试验结果。对ECOG 4599实验的进一步亚组分析发现，腺癌患者接受贝伐单抗联合紫杉醇/卡铂方案比单纯化疗可更显著改善生存（14.2个月 *vs* 10.03个月，*P*=0.04)^[21]^。最近一项系统回顾和荟萃分析^[22]^也表明，贝伐单抗联合以铂类为基础化疗方案一线治疗晚期NSCLC能够明显延长患者OS和PFS，且在腺癌及体重下降小于5%的患者中更为明显。但是到目前为止，尚无标志物能预测腺癌患者中哪些群体能从贝伐单抗中获益更多。因此NCCN推荐贝伐单抗联合化疗可作为一种治疗选择，用于体能状态（performance status, PS）评分为0分-1分，无*EGFR*突变的非鳞状细胞癌。

*KRAS*突变约占北美肺腺癌患者的25%，是最为常见的突变类型。肺腺癌中大部分*KRAS*突变与吸烟有关。*KRAS*突变主要见于腺癌，是EGFR-TKI反应的负性预测因子，而与化疗疗效无关。*KRAS*突变与*EGFR*突变相互排斥，携带*KRAS*突变的患者不宜采用EGFR-TKI治疗。目前针对*KRAS*突变的靶向治疗正处于临床试验阶段，Janne等^[23]^公布了一项前瞻性、多中心、双盲、安慰剂、随机对照的Ⅱ期临床试验，首次证明了MEK抑制剂司美替尼（selumetinib）联合多西他赛二线治疗*KRAS*突变的晚期NSCLC有较佳疗效。这又为*EGFR*野生型患者中*KRAS*突变阳性的患者带来了一种新的治疗选择。

### 2.2 二线、三线治疗选择

单药多西他赛、培美曲塞（非鳞癌）及厄洛替尼（*EGFR*突变阳性）是目前的标准二线治疗方案。既往的研究证明，在未经选择的NSCLC二、三线治疗中，厄洛替尼优于最佳支持治疗^[24]^，且与培美曲塞疗效相当^[25]^，达到了二线治疗标准。但对于*EGFR*野生型患者，厄洛替尼能否用于二、三线治疗尚无定论。几项Ⅱ期临床试验^[26-28]^结果均表明，厄洛替尼治疗复治的*EGFR*野生型患者具有一定疗效。Jazieh等^[29]^对厄洛替尼治疗*EGFR*野生型NSCLC的相关研究（BR.21、TITAN等）进行了回顾性分析，结论认为厄洛替尼可以作为*EGFR*野生型患者的二线、三线治疗选择。中山大学肿瘤防治中心的一项Ⅱ期随机临床试验^[30]^表明，厄洛替尼对比培美曲塞二线治疗晚期*EGFR*突变型及野生型肺腺癌，二者疗效无差异。然而一项比较厄洛替尼与多西他赛治疗*EGFR*野生型患者的前瞻性研究（TAILOR）^[31]^提示多西他赛较厄洛替尼明显改善PFS，并且显著提高缓解率与疾病控制率，厄洛替尼疗效有限。另一项比较厄洛替尼与多西他赛二线治疗NSCLC的Ⅲ期随机试验（DELTA）^[32]^也表明，*EGFR*野生型亚组中，多西他赛较厄洛替尼明显延长PFS，但未转化为生存优势。因此目前的研究表明，多西他赛和培美曲塞是*EGFR*野生型患者的标准二线、三线治疗方案，而厄洛替尼也能作为该类患者的二线、三线治疗选择，尤其适用于PS评分较差，不能承受化疗的患者。

一项克唑替尼对比单药多西他赛或培美曲塞，二线治疗ALK阳性的晚期NSCLC的Ⅲ期开放临床试验^[33]^数据表明，克唑替尼对比化疗延长了PFS（7.7个月 *vs* 3.0个月，*P*＜0.001），具有更高的ORR（65% *vs* 20%, *P*＜0.001），同时能更好地控制疾病相关的症状，提高生活质量，但OS无差异。该数据表明，对于*EGFR*野生型患者中的ALK阳性患者，二线治疗可以考虑选择克唑替尼。

目前尚无证据证明联合化疗对比单药化疗更有优势。关于靶向联合治疗，近期的一项系统回顾性研究表明，靶向药物联合对比单药厄洛替尼二线治疗NSCLC，OS、PFS、ORR均有获益，进一步的亚组分析表明，EGFR阴性及KRAS阳性患者似乎更倾向于联合靶向治疗。但该回顾性分析中包含的单项研究结果均不支持靶向联合治疗，理由是联合治疗没有延长OS，且毒性增加。

### 2.3 维持治疗

对于晚期NSCLC在经历4个-6个周期的化疗后，如肿瘤缓解或稳定，可以考虑给予继续维持治疗或换药维持治疗。

维持治疗，在*EGFR*野生型的非鳞状细胞癌，贝伐单抗可以在4个-6个周期的初始治疗（即，含铂两药化疗联合贝伐单抗）之后继续使用^[34]^；培美曲塞也能作为*EGFR*野生型NSCLC的维持治疗，近期的一个Ⅲ期随机临床试验（PARAMOUNT）^[35]^发现培美曲塞对比安慰剂的维持治疗增加了患者的PFS（4.1个月 *vs* 2.8个月），延长了OS（13.9个月 *vs* 11.0个月，*P*=0.019, 5）；最近的POINTBREAK试验^[36]^的研究数据表明贝伐单抗+培美曲塞相比单药培美曲塞对该类患者视乎更有优势（PFS：6个月 *vs* 5.6个月），但OS无差异，而AVAPERL（MO22089）试验^[37]^则表明贝伐单抗+培美曲塞相比单药贝伐单抗PFS明显延长（7.4个月 *vs* 3.7个月），OS也有所延长（17.1个月 *vs* 13.2个月），但无统计学意义。在*EGFR*野生型的NSCLC，单药西妥昔单抗也可用于4个-6个周期的初始治疗（即，顺铂+Ⅲ长春瑞滨联合西妥昔单抗）后的维持治疗^[38]^；一项Ⅲ期随机临床试验^[39]^对比了吉西他滨+顺铂一线化疗后以吉西他滨或厄洛替尼维持治疗的差异，结果显示，与观察组（1.9个月）相比，吉西他滨组（3.8个月）PFS延长程度大于厄洛替尼组（2.9个月）。因此吉西他滨也可作为*EGFR*野生型NSCLC维持治疗的一种选择。

关于换药维持治疗，2项Ⅲ期随机临床试验^[40, 41]^表明，对于一线治疗4个-6个周期后无进展患者，开始培美曲塞或厄洛替尼维持治疗能带来PFS和OS获益。因此培美曲塞可以用于*EGFR*野生型的非鳞状细胞癌的换药维持治疗，而厄洛替尼则用于有或没有*EGFR*突变的非鳞状细胞癌或鳞癌患者。此外多西他赛也被证明可用于鳞状细胞NSCLC患者的换药维持治疗。

## 3 *EGFR*野生型NSCLC疗效预测标志物

目前，许多分子标志物被用来预测NSCLC化疗疗效。如切除修复交叉互补基因（excision repair cross-complementation group 1, ERCC1）表达水平可以用于预测含铂化疗方案治疗NSCLC的疗效^[42]^。核糖核苷还原酶调节因子1（ribonucleotide reductase M1, RRM1）表达水平与吉西他滨为基础的化疗疗效相关^[43]^。因此学者们试图寻找具有预测作用的分子标记物，以提高*EGFR*野生型患者的化疗疗效。黄诚等^[44]^研究表明，以ERCC1、RRM1、TS作为分子标记物指导*EGFR*野生型患者化疗，能显著提高患者的PFS。

尽管*EGFR*野生型对EGFR-TKI反应欠佳，但仍有部分患者能从中获益。VeriStrat测试是利用基质辅助激光解析电离质谱分析方法进行基于患者血清或血浆的检测，得到“VS-G”或“VS-P”的结果，用来预测EGFR-TKI的疗效。2013年ASCO报道的一项Ⅲ期临床研究PROSE^[45]^证实，通过VeriStrat评分可以有效指导*EGFR*野生型及突变状态未知的患者二线治疗选择单药化疗还是厄洛替尼。

## 4 结论

目前的研究表明*EGFR*野生型患者的治疗首选化疗。*EGFR*突变状态与化疗疗效相关，但突变状态是否能决定化疗方案的选择有待进一步研究。靶向治疗或靶向联合化疗方案，如克唑替尼、贝伐单抗联合紫杉醇+卡铂、西妥昔单抗/顺铂/长春瑞滨等为*EGFR*野生型患者提供了更多治疗选择。*EGFR*野生型NSCLC的二线、三线治疗首选单药多西他赛或培美曲塞，而厄洛替尼也可考虑用于该类患者的二线、三线治疗，尤其适用于PS评分较差，不能承受化疗的患者。贝伐单抗、培美曲塞均可用于*EGFR*野生型患者的维持治疗，而贝伐单抗联合培美曲塞显示了优于两者单用的疗效，此外，西妥昔单抗、吉西他滨、厄洛替尼、多西他赛等也可尝试用于*EGFR*野生型患者的维持治疗。ERCC1、RRM1、TS等具有预测作用的分子标记物，可以提高*EGFR*野生型患者的化疗疗效。VeriStrat测试评分则可以有效指导*EGFR*野生型患者二线治疗选择单药化疗还是厄洛替尼。*EGFR*野生型在NSCLC中占大多数，因此需要发展新战略提高野生型患者疗效，如寻找新靶点及研发新的靶向药物、寻找新的分子预测标志物、探索靶向联合阻断相关通路等。期待更多的研究和临床试验为*EGFR*野生型患者找到最佳的治疗选择。
